# Interleukin-4 Protects Dopaminergic Neurons *In vitro* but Is Dispensable for MPTP-Induced Neurodegeneration *In vivo*

**DOI:** 10.3389/fnmol.2017.00062

**Published:** 2017-03-09

**Authors:** Laura Hühner, Jennifer Rilka, Ralf Gilsbach, Xiaolai Zhou, Venissa Machado, Björn Spittau

**Affiliations:** ^1^Department of Molecular Embryology, Institute for Anatomy and Cell Biology, Faculty of Medicine, University of FreiburgFreiburg, Germany; ^2^Institute of Experimental and Clinical Pharmacology and Toxicology, University of FreiburgFreiburg, Germany; ^3^Department of Molecular Biology and Genetics, Weill Institute for Cell and Molecular Biology, Cornell University, IthacaNY, USA

**Keywords:** IL4, MPTP, mDA neuron, microglia, neurodegeneration

## Abstract

Microglia are involved in physiological as well as neuropathological processes in the central nervous system (CNS). Their functional states are often referred to as M1-like and M2-like activation, and are believed to contribute to neuroinflammation-mediated neurodegeneration or neuroprotection, respectively. Parkinson’s disease (PD) is one the most common neurodegenerative disease and is characterized by the progressive loss of midbrain dopaminergic (mDA) neurons in the substantia nigra resulting in bradykinesia, tremor, and rigidity. Interleukin 4 (IL4)-mediated M2-like activation of microglia, which is characterized by upregulation of alternative markers Arginase 1 (Arg1) and Chitinase 3 like 3 (Ym1) has been well studied *in vitro* but the role of endogenous IL4 during CNS pathologies *in vivo* is not well understood. Interestingly, microglia activation by IL4 has been described to promote neuroprotective and neurorestorative effects, which might be important to slow the progression of neurodegenerative diseases. In the present study, we addressed the role of endogenous and exogenous IL4 during MPP^+^-induced degeneration of mDA neurons *in vitro* and further addressed the impact of IL4-deficiency on neurodegeneration in the 1-methyl-4-phenyl-1,2,3,6-tetrahydropyridine (MPTP) mouse model of PD *in vivo*. Our results clearly demonstrate that exogenous IL4 is important to protect mDA neurons *in vitro*, but endogenous IL4 seems to be dispensable for development and maintenance of the nigrostriatal system as well as MPTP-induced loss of TH^+^ neurons *in vivo*. These results underline the importance of IL4 in promoting a neuroprotective microglia activation state and strengthen the therapeutic potential of exogenous IL4 for protection of mDA neurons in PD models.

## Introduction

Microglia are the resident immune cells of the central nervous system (CNS), and thus are involved in a plethora of physiological as well as neuropathological conditions (Prinz and Priller, 2014). Similar to peripheral macrophages (Mosser, 2003; Gordon and Martinez, 2010), the functions of microglia are regulated by various endogenous and exogenous stimuli and their functional states are referred to as M1-like and M2-like activation (Prinz and Priller, 2014). M1-like microglia activation induced by Th1 cytokines, such as IFNγ or the bacterial lipopolysaccharide (LPS), induces secretion of pro-inflammatory cytokines and reactive oxygen species, which are believed to contribute to neuroinflammation-mediated neurodegeneration (Block et al., 2007). M2-like microglia activation is induced by the Th2 cytokines Interleukin 4 (IL4) and Interleukin 13 (IL13) and characterized by upregulation of alternative activation markers Arginase-1 (Arg1) and Chitinase 3 like 3 (Ym1) (Colton and Wilcock, 2010; Zhou et al., 2012). IL4-induced M2-like microglia/macrophage activation has been described to exert neuroprotective effects in mouse models for spinal cord injury (Francos-Quijorna et al., 2016), cerebral ischemia (Liu et al., 2016) and multiple sclerosis (Butti et al., 2008). Next to the abovementioned CNS pathologies, M2-like microglia activation has been further described in animal models of Parkinson’s disease (PD) and has been associated with reduced progression and disease severity (Moehle and West, 2015). PD is the second most common neurodegenerative disease and is characterized by the progressive loss of midbrain dopaminergic (mDA) neurons in the substantia nigra (SN) (Jellinger, 2001). This loss of mDA neurons results in a subsequent reduction in striatal dopamine (DA) levels, which is responsible for the classical clinical symptoms such as resting tremor, bradykinesia and rigidity (Jankovic, 2008). Although the IL4-mediated activation of microglia, which is characterized by upregulation of alternative markers Arginase 1 (Arg1) and Chitinase 3 like 3 (Ym1) has been well studied *in vitro* (Zhou et al., 2012), the role of endogenous IL4 during CNS pathologies *in vivo* is not well understood. IL4-deficient mice have been reported to develop normally, without showing morphological abnormalities, reduced secretion of IL5 and IL10, as well as an impaired expression of IL4-dependent IgE and IgG_1_ (Metwali et al., 1996). Moreover, loss of IL4 resulted in decreased neuron-microglia communication via the CD200-CD200R ligand-receptor pair and increased neuroinflammatory responses after LPS application (Lyons et al., 2009). CNS-derived IL4 has been further shown to play important roles during regulation of microglia/macrophage activation in the EAE model for multiple sclerosis (Ponomarev et al., 2007). In the present study, we have analyzed the role of endogenous and exogenous IL4 during MPP^+^-induced degeneration of mDA neurons *in vitro* and further addressed the impact of IL4-deficiency on neurodegeneration in the 1-methyl-4-phenyl-1,2,3,6-tetrahydropyridine (MPTP) mouse model of PD *in vivo*. Our results clearly demonstrate that IL4 is important to protect mDA neurons *in vitro* but seems to be dispensable for MPTP-induced loss of TH^+^ neurons *in vivo*.

## Materials and Methods

### Animals

C57BL/6 mice were housed at 22 ± 2°C under a 12 h light/dark cycle with *ad libitum* access to food and water. C57BL/6-IL4^tm1Nnt^/J mice as described by Metwali et al. (1996) were obtained from the Jackson Laboratory (via Charles River, Germany) and are from now on referred to as IL4 KO mice. All animal procedures were conducted in strict accordance with the German federal animal welfare law, local ethical guidelines and have been approved by the animal experimentation committee of the University of Freiburg and the Regierungspräsidium Freiburg (G-11/77 [MPTP], X-12/01D [E14 mDA neurons], X-12/02D [primary microglia]).

### MPTP-Induced Neurodegeneration

Adult (10–12 weeks) male wild type (WT) and IL4 KO mice were intraperitoneally injected with 20 mg/kg MPTP hydrochloride (Sigma-Aldrich) dissolved in 0.2 ml phosphate-buffered saline (PBS) once a day for three consecutive days as previously described (Machado et al., 2016a). Mice were sacrificed after 1, 2, 7, and 90 days after injections. PBS only injected mice served as controls. A total of 64 mice were used throughout this study (WT *n* = 37, IL4 KO *n* = 27). All procedures involving MPTP were conducted in strict accordance with published safety and handling guidelines (Przedborski et al., 2001).

### Primary Microglia Cultures

Primary microglia cultures were generated as previously described (Spittau et al., 2013). Briefly, blood vessels and meninges were removed from brains of P0/P1 C57BL/6 mice (Janvier) and brains were washed in ice-cold Hank’s BSS (Gibco, Germany). After enzymatic dissociation with Trypsin-EDTA (Gibco, Germany) for 15 min at 37°C, an equal volume of fetal calf serum (FCS, Gibco, Germany) together with DNase (Roche, Mannheim, Germany) at a final concentration of 0.05 mg/ml was added. Cells were dissociated using wide- and narrow-bored polished Pasteur pipettes. Finally, cells were centrifuged and resuspended in DMEM/F12 medium (Gibco, Germany) containing 10% FCS and 1% penicillin/streptomycin (Invitrogen). Cell suspensions from 2–3 brains were plated on poly-D-lysine-coated (Sigma-Aldrich, Schnelldorf, Germany) 75 cm^2^ culture flasks. Cultures were kept in a 5% CO_2_/95% humidified atmosphere at 37°C. After 10–14 days in culture, microglia were shaken off (250–300 rpm for 1 h) from adherent astrocytes and were plated according to the experimental purposes.

### E14 Ventral Midbrain-Derived Neuron-Enriched and Mixed Neuron-Glia Cultures

Neuron-enriched and mixed neuron-glia cultures from embryonic day 14 (E14) ventral midbrains of NMRI mice (Charles River Laboratories Inc.) were generated as described previously (Machado et al., 2016b). Dissected ventral midbrains were dissociated using Trypsin-EDTA (Gibco) for 15 min at 37°C. DNase (Roche) was added to a final concentration of 0.05 mg/ml and after gentle trituration using fire-polished Pasteur pipettes, cell suspensions were centrifuged at 1000 × *g* for 4 min. Cell pellets were resuspended in DMEM/F12 medium (Gibco) containing either 10% FCS, 10% horse serum (HS) and 1% penicillin/streptomycin (Invitrogen) to obtain mixed neuron-glia cultures, or N2 supplement (Invitrogen) and 1% penicillin/streptomycin (Invitrogen) to obtain neuron-enriched cultures. Both cultures were plated on poly-D-lysine-coated (Sigma-Aldrich) glass coverslips (12 mm diameter) at a density of 1–2 midbrains/coverslip. On day *in vitro* 1 (DIV1) medium was refreshed and mixed neuron-glia cultures were maintained for additional 7 days to yield mature cultures. Neuron-enriched cultures were treated from DIV1 according to the experimental design. Cultures were kept in a 5% CO_2_/95% humidified atmosphere at 37°C. MPP^+^ (Sigma-Aldrich) treatments were performed as described recently (Spittau et al., 2012). IL4 (10 ng/ml) and IGF-1 (50 ng/ml) were obtained from Peprotech (Germany) and prepared and dissolved according to the manufacturer’s instructions.

### Neutralization of IL4 *In vitro*

Blocking of IL4 in E14 ventral midbrain mixed neuron-glia cultures was achieved using a IL4 neutralizing antibody (IL4nAB) obtained from Ebiosciences (Frankfurt, Germany). Functionality and optimal concentrations were confirmed in IL4-treated primary microglia cultures and the IL4 nAB was used in mixed neuron-glia cultures at a dilution of 1:50.

### RNA Isolation and Reverse Transcription

Total RNA was isolated from primary microglia using TRIzol reagent (Invitrogen, Karlsruhe, Germany) according to manufacturer’s instructions. RNAs from SN and caudate putamen (CPu) were isolated after dissection and transfer to RNA later (Ambion). Tissues were homogenized in peqGOLD TriFast (PeqLab) using a Precellys 24 homogenizer (PeqLab). RNA concentration and quality was determined using the NanoDrop 2000 (Thermo Scientific, Germany). 1 μg RNA from each sample was reverse transcribed to cDNA using RevertAid (Fermentas, St. Leon-Rot, Germany) according to the manufacturer’s instructions.

### Semiquantitative and Quantitative RT-PCR

Semiquantitative RT-PCR (Eppendorf Mastercycler, Eppendorf, Germany) was performed using the PCR Red-Mastermix 2x (Genaxxon Bioscience, Ulm, Germany). PCR products were separated using agarose gel electrophoresis and visualized after staining with GelRed (Genaxxon Bioscience). Images were captured using a Biometra (Göttingen, Germany) gel documentation station. Quantitative RT-PCR was performed using the MyiQ^TM^ system (Bio-Rad, München, Germany) in combination with the Quantitect SYBR Green PCR Kit (Applied Biosystems, Darmstadt, Germany). 1 μl of cDNA template was used in 25 μl reaction mixture. Results were analyzed using the Bio-Rad iQ5 Optical System Software and the comparative CT method. All data are expressed as 2^-ΔΔCT^ for the gene of interest normalized to the housekeeping gene Gapdh and presented as fold change relative to controls. The following primers have been used throughout this study: Arg1*for* 5′- AACACTCCCCTGACAACCAG-3′, Arg1*rev* 5′-CTGAAAGGAGCCCTGTCTTG-3′ [NM_007482.3], Igf1*for* 5′-CTGGACCAGAGACCCTTTGC-3′, Igf1*rev* 5′-GGACGGGGACTTCTGAGTCTT-3′ [NM_010512], IL4f*or* 5′-ATTTTGAACGAGGTCACAGGAGAAG-3′, IL4*rev* 5′-ACCTTGGAAGCCCTACAGACGAG-3′ [NM_021283.2], Gapdh*for* 5′-GGCATTGCTCTCAATGACAA-3′, Gapdh*rev* 5′-ATGTAGGCCATGAGGTCCAC-3′[NM_001289726].

### ELISA

Interleukin 4 was detected in serum-free culture medium from E14 mixed neuron-glia cultures from control and MPP^+^-treated groups after 48 h as well as in medium from primary microglia cultures from control and MPP^+^-treated groups after 24 h using the murine IL4 ELISA Development Kit (Peprotech, Hamburg, Germany). IGF-1 was detected in serum-free culture medium from control IL4-treated primary microglia cultures using the murine IGF-1 ELISA Development Kit (Peprotech). ELISAs were performed according to the manufacturer’s instructions. Color reactions were performed using 2,2 azino-bis(3-ethylbenzothiazoline-6-sulphonic acid) substrate (ABTS, Sigma-Aldrich, Germany) for 30 min in the dark. Finally, absorbance was detected using Multiskan FC plate reader (Thermo Fischer) at the absorption of 405 nm. Concentrations of IL4 and IGF-1 were calculated from standard curves using the GraphPad Prism5 software (GraphPad Software Inc.).

### Immunohistochemistry and Immunocytochemistry

Numbers of tyrosine hydroxylase (TH) positive neurons in E14 ventral midbrain neuron-enriched as well as mixed neuron-glia cultures were determined after immunocytochemistry. After incubation of neuron cultures for different time points, coverslips were washed once with PBS and fixed using 4% paraformaldehyde in PBS for 15 min at room temperature (RT). Cells were washed three times with PBS for 5 min each, and subsequently blocked and permeabilised for 1 h at RT in PBS containing 10% normal goat serum (Invitrogen, Darmstadt, Germany) and 0.1% Triton-X-100 (Roche Diagnostics). Primary antibodies rabbit anti-TH (1:1.000, polyclonal, Millipore, Schwalbach, Germany), anti-Iba1 (1:500, polyclonal, Wako) and anti-Gfap (1:800, monoclonal, Millipore) were incubated overnight at RT. Afterward, coverslips were washed three times with PBS, and cells were incubated with goat-anti-rabbit peroxidase-conjugated secondary antibodies (1:500, Nordic Immunology) for 1 h at RT. The immunoreactivity was visualized using diaminobenzidine (Sigma-Aldrich, Deisenhofen, Germany) as described by Adams (1981). Tile scans from whole coverslips were obtained using an Zeiss AxioImager M2 (Zeiss, Göttingen, Germany) and cell counting was performed using ImageJ software (NIH). Immunofluorescent stainings were performed using fixed cultures on glass coverslips as mentioned above, followed by incubation with goat anti-mouse or goat anti-rabbit fluorescence-coupled secondary antibodies (1:200, Cell Signaling Technologies) for 1 h at RT. The sections were then washed three times with PBS for 3 min each and nuclei were counterstained using 4′,6-diamidino-2-phenylindole (DAPI, Roche). After a final washing step, sections were placed on objective slides and mounted with Fluoromount G mounting medium (SouthernBiotech). Fluorescence images were captured using the Zeiss AxioImager M2 (Zeiss, Göttingen, Germany).

### Unbiased Stereology

Numbers of TH^+^ mDA neurons, Iba1^+^ microglia and Gfap^+^ astrocytes were estimated according to the optical fractionator methods using the Stereo Investigator software (MBF Bioscience, Germany). Cell counting was performed using an oil immersion 63 × objective (AxioImager M1, Zeiss, Göttingen, Germany), counting frame of 50 μm × 50 μm and a grid size of 120 μm × 100 μm. The optical fractionator was optimized to reach a coefficient of error ≤0.1. For each animal four sections with an evaluation interval of three were selected and labeled cells were counted. The nucleus of each immunoreactive cell was chosen as the counting event. Cell numbers are either presented as total estimated cell numbers or percentages (MPTP model) compared to PBS-treated control animals.

### HPLC-Based Quantification of Striatal Dopamine Levels

Dopamine levels were quantified as recently described (Machado et al., 2016a). Striatal tissues were dissected (Bregma 1.32-0) at 7 and 90 days after MPTP injections, weighted and transferred to ice-cold 0.2 M perchloric acid (40 μl/mg tissue). Samples were homogenized in 0.2 M perchloric acid using a Precellys 24 tissue homogenizer (Peqlab, Germany). HPLC-ED was performed as described recently (Schneider et al., 2011). Briefly, homogenates were centrifuged at 20.000 × *g* for 5 min at 4°C and filtered through 0.2 μm filters. DA concentrations were detected using a HPLC pump (UltiMate 3000 Quaternary Analytical, Dionex), an autosampler (WISP 717plus, Waters, Germany) and an amperometric detector (Antec Intro, Antec Leyden, Netherlands). The isocratic mobile phase consisted of sodium acetate (90 mM), citric acid monohydrate (60 mM), ethylene- diaminetetraacetic acid (2 mM), 1-octanesulfonic acid (3 mM), and 8% methanol and was adjusted to a pH of 5.3. DA was separated on a Prontosil 120-3-C18 AQ column (3 μm, 120 × 2 mm; Bischoff chromatography, Germany) by using a fixed flow rate of 300 μl/min. Data were registered and analysed by using Chromeleon 6 software (Dionex).

### Statistics

Data are given as means ± standard error of the mean (SEM). Statistical differences between two groups were determined using Student’s *t*-test. Normality of samples was assessed using the D’Agostino and Pearson omnibus normality test (as recommended by GraphPad Prism) and non-parametric tests have been used where appropriate. Multiple-group analysis was performed using one-way ANOVA followed by Bonferroni’s multiple comparison post-test. *P*-values ≤0.05 were considered as statistically significant. All statistical analyses were performed using the GraphPad Prism5 software (GraphPad Software Inc.).

## Results

### IL4 Protects mDA Neurons from MPP^+^-Induced Degeneration in the Presence of Glia Cells

**FIGURE 1:**
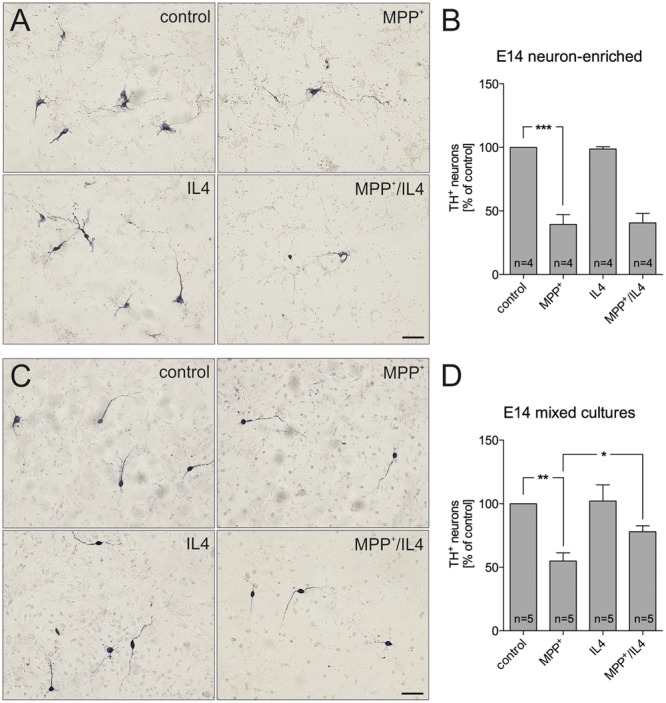
**Interleukin 4 (IL4) protects midbrain dopaminergic (mDA) neurons from MPP^+^-induced neurodegeneration in mixed neuron-glia cultures.** E14 ventral midbrain neuron-enriched cultures **(A)** and E14 ventral midbrain mixed neuron-glia cultures **(C)** were treated with 0.2 μM MPP^+^ in the presence or absence of IL4 (10 ng/ml) for 2 days and numbers of TH^+^ neurons were counted. Scale bars indicate 50 μm. Quantifications and statistical evaluations revealed that IL4 failed to protect mDA neurons in E14 neuron-enriched cultures **(B)** but was sufficient to significantly protect mDA neurons in E14 mixed neuron-glia cultures **(D)**. Data are given as mean ± SEM from four (neuron-enriched cultures) and five (neuron-glia cultures) independent experiments performed in duplicates. *P*-values derived from one-way ANOVA followed by Bonferroni’s multiple comparison post-test are ^∗^*p* < 0.05, ^∗∗^*p* < 0.01 and ^∗∗∗^*p* < 0.001.

In order to address the neuroprotective potential of IL4 against MPP^+^-induced mDA neurodegeneration, E14 ventral midbrain cultures were treated for 48 h with MPP^+^ (0.2 μM) in the absence or presence of recombinant IL4 (10 ng/ml). **Figures 1A,B** demonstrates that neuron-enriched cultures responded to MPP^+^ application with a significant loss of mDA neurons (39.39% ± 7.821). IL4 co-treatment was not sufficient to protect mDA neurons in neuron-enriched cultures from MPP^+^-induced neurodegeneration (40.59% ± 7.417). Next, mDA neurodegeneration was induced by MPP^+^ in mixed neuron-glia cultures. As shown in **Figures 1C,D**, MPP^+^ treatment alone resulted in a significant decrease of mDA neurons in mixed neuron-glia cultures (54.93% ± 6.361). Interestingly, IL4 co-treatment significantly increased the survival of mDA neurons in mixed neuron-glia cultures (77.98% ± 4.655). These results indicate that IL4 only exerts its neuroprotective effects against MPP^+^-induced neurodegeneration in the presence of glia cells.

### Neutralization of IL4 Increases MPP^+^-Induced Neurodegeneration *In vitro*

**FIGURE 2:**
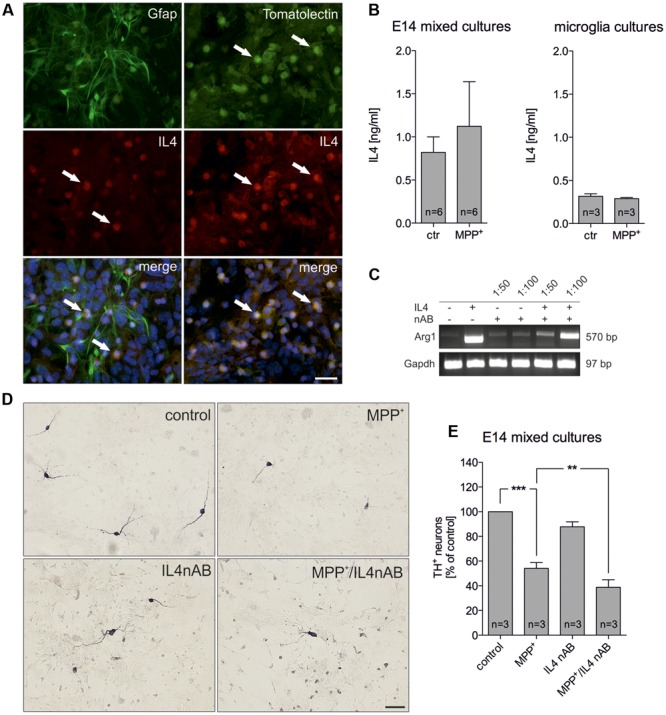
**Neutralization of endogenous IL4 increases MPP^+^-induced neurodegeneration *in vitro*. (A)** Cellular expression of IL4 in E14 mixed neuron-glia cultures was determined after co-staining with Gfap as an astrocyte marker and FITC-coupled tomato lection as a microglia marker. White arrows mark IL4^+^ cells. Scale bar indicates 30 μm. **(B)** Secretion of IL4 in E14 mixed neuron-glia cultures under control conditions and 2 days after MPP^+^ treatment was determined using an IL4 ELISA. The levels of secreted IL4 in supernatants of primary microglia cultures 24 h after MPP^+^ treatment (0.2 μM) was analyzed using an IL4 ELISA. **(C)** Validation of IL4 neutralization *in vitro*. Primary microglia cultures were treated with recombinant IL4 (10 ng/ml) in the presence (1:50 and 1:100) or absence of an IL4 neutralizing antibody and IL4-mediated increase of Arg1 expression was assessed by PCR. Gapdh was used as housekeeping gene. **(D)** E14 ventral midbrain mixed neuron-glia cultures were treated with 0.2 μM MPP^+^ in the presence or absence of an IL4 neutralizing antibody (IL4nAB) at a dilution of 1:50 for 2 days and numbers of TH^+^ neurons were counted. Scale bars indicate 50 μm. **(E)** Quantifications and statistical evaluation demonstrates that neutralization of IL4 increases MPP^+^-induced degeneration of mDA neurons *in vitro*. Data are given as mean ± SEM from six **(B)** and three **(D,E)** independent experiments performed in duplicates. *P*-values derived from one-way ANOVA followed by Bonferroni’s multiple comparison post-test are ^∗∗^*p* < 0.01 and ^∗∗∗^*p* < 0.001.

Since IL4 has been recently described to be expressed by microglia (Park et al., 2005), we analyzed whether IL4 is expressed in the used E14 mixed neuron-glia culture system. Whereas Gfap^+^ astrocytes were negative for IL4, microglia labeled with FITC-coupled tomato lectin displayed a strong immunoreactivity for IL4 (**Figure 2A**). Using an IL4 ELISA, we further analyzed the levels of IL4 in supernatants of control- and MPP^+^-treated cultures. **Figure 2B** demonstrates that 0.821 ng/ml (± 0.179) IL4 was detectable after 48 h in supernatants from control cultures and a slight but not significant increase of IL4 levels (1.123 ng/ml ± 0.5164) could be determined after treatment with MPP^+^. Moreover, we addressed whether primary microglia respond to MPP^+^ treatment with changes in IL4 secretion. 24 h after treatment with MPP^+^ no change in IL4 secretion from primary microglia cultures was detectable. Interestingly, the data demonstrate that microglia in mixed neuron-glia cultures release higher levels of IL4 than primary microglia. We next aimed to inhibit endogenous IL4 in mixed-neuron glia cultures in order to determine the role of microglia-derived IL4 during MPP^+^-induced neurodegeneration. Thus, the functionality of a neutralizing IL4 antibody (IL4 nAB) was validated. Therefore, primary microglia cultures were treated with recombinant IL4 (10 ng/ml) in the presence or absence of IL4 nAB at dilutions of 1:100 and 1:50, respectively. Using semiquantitative RT-PCR, expression of the IL4-induced M2 marker Arg1 was analyzed. As shown in **Figure 2C**, Arg1 expression was induced after treatment of primary microglia with IL4 for 24 h. Almost complete abrogation of IL4-triggered Arg1 expression was observed in the presence of the IL4 nAB at a dilution of 1:50, which was used for further IL4 neutralization experiments. Next, mixed-neuron glia cultures were treated with MPP^+^ and endogenous IL4 was blocked (IL4 nAB, 1:50). **Figures 2D,E** show that inhibition of endogenous IL4 resulted in a significant increase in MPP^+^-induced neurodegeneration (38.75 % ± 6.116) as compared to MPP^+^ treatment alone (54.08% ± 4.72). These data clearly demonstrate that microglia-derived IL4 in mixed-neuron glia cultures is important to protect mDA neurons from MPP^+^ intoxication.

### Conditioned Medium from IL4-Treated Primary Microglia Promotes Survival of mDA Neurons

**FIGURE 3:**
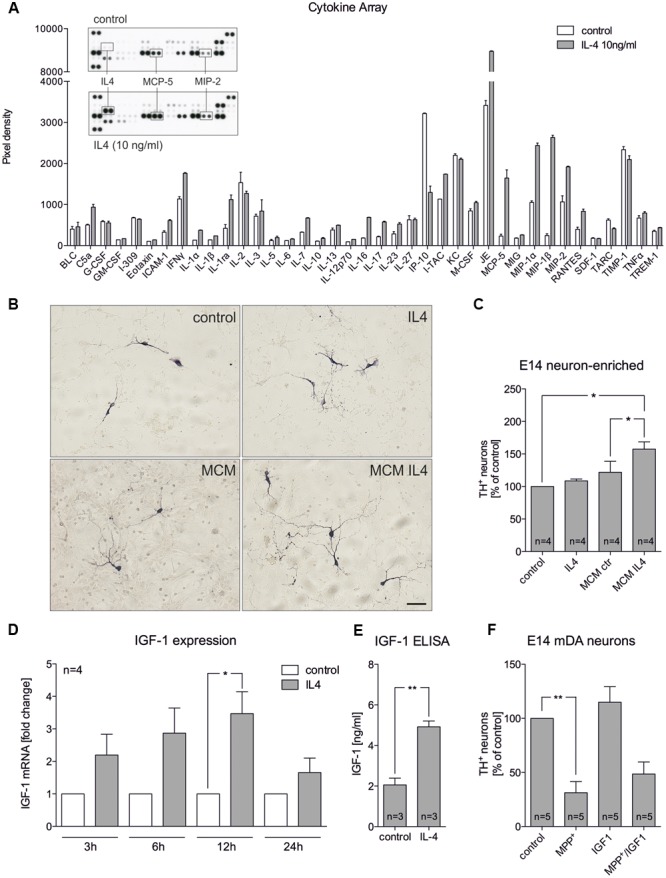
**Microglia-conditioned medium after IL4 treatment is neuroprotective *in vitro*. (A)** Treatment of primary microglia with recombinant IL4 (10 ng/ml) for 24 h results in modest changes of secreted chemokines and cytokines. As expected, IL4 was detected in IL4-treated samples and thus was not included in the densitometric spot analysis. Levels of MCP-5 and MIP-2 were increased after IL4 treatment. Data are given as mean ± SEM from two independent experiments. **(B)** E14 ventral midbrain neuron-enriched cultures were treated for 2 days with serum-free medium (control) and IL4 (10 ng/ml) or with microglia conditioned medium obtained after treatment of primary microglia for 24 h with serum-free medium (MCM) and IL4 at 10 ng/ml (MCM IL4). Scale bar indicates 50 μm. **(C)** Quantifications of TH^+^ neurons indicate that microglia conditioned medium after treatment with IL4 (MCM IL4) significantly increased neuron survival. IL4 alone was not able to promote neuroprotection. **(D)** IL4 treatment increases IGF-1 expression in primary microglia. After treatment for 3, 6, 12, and 24 h IGF-1 expression was determined using qPCR and is presented as fold change compared to untreated control cultures. Significant increases in IGF-1 expression was observed after 12 h. **(E)** IGF-1 secretion under control conditions and 24 h after treatment with IL4 (10 ng/ml) was detected using an IGF-1 ELISA. **(F)** Recombinant IGF-1 (50 ng/ml) increased neuron survival in E14 ventral midbrain neuron-enriched cultures without reaching significance. Data are given as mean ± SEM from four **(B–D)**, three (**C**, ELISA) and five **(E)** independent experiments performed in duplicates. *P*-values derived from student’s *t*-test **(D)** are ^∗^*p* < 0.05 and ^∗∗^*p* < 0.01. *P*-values derived from one-way ANOVA followed by Bonferroni’s multiple comparison post-test are ^∗^*p* < 0.05 and ^∗∗^*p* < 0.01.

The abovementioned results indicate that IL4 might shape the functions and properties of microglia in mixed neuron-glia cultures to promote survival of mDA neurons. Hence, primary microglia were treated with IL4 and secretion of cytokines and chemokines were analyzed. As shown in **Figure 3A**, results of the array using the proteome profiler kit for inflammatory cytokines revealed that IL4 did not influence the secretion of the vast majority of cytokines/chemokines from microglia. Notably, MCP-5 and MIP2 were two candidates with increased secretion after IL4 treatment (10 ng/ml) for 24 h. We further used microglia-conditioned medium to treat E14 neuron-enriched cultures in order to determine the survival promoting effects of microglia-derived factors on mDA neurons. **Figures 3B,C** show that microglia-conditioned medium after treatment with IL4 for 24 h (MCM IL4) significantly increased mDA neuron survival (157.4% ± 11.13) compared to microglia control medium (MCM). The observed neuroprotective effect of MCM IL4 was not promoted by IL4 itself, since direct IL4 treatment of E14 neuron-enriched cultures did not result in an increase of surviving TH^+^ neurons (108.5% ± 2.849). This observation demonstrates that IL4 treatment of microglia induces the secretion of neuroprotective factors, which increase the survival of TH^+^ neurons in E14 neuron-enriched cultures. IGF-1 has been identified as a survival-promoting factor secreted by macrophages after IL4 treatment (Wynes et al., 2004). Therefore, we analyzed whether IL4 treatment of primary microglia increases expression and secretion of IGF-1. Using quantitative RT-PCR, we observed that IGF-1 mRNA levels increased after IL4 treatment, reaching a significant peak after 12 h of treatment (**Figure 3D**). Moreover, **Figure 3E** clearly demonstrates that secretion of IGF-1 from primary microglia was significantly increased after 24 h (4.919 ng/ml ± 0.2868) compared to untreated control cells (2.061 ng/ml ± 0.3321). As IL4 was able to protect TH^+^ neurons from MPP^+^-induced degeneration in mixed neuron-glia cultures, we wanted to address whether microglia-derived IGF-1 was responsible for this protective effect. Thus, recombinant IGF-1 was used in order to determine whether TH^+^ neurons in E14 neuron-enriched cultures could be protected from MPP^+^-induced neurodegeneration. As shown in **Figure 3F**, MPP^+^ treatment significantly reduced the numbers of TH^+^ neurons (38.2% ± 9.848) and a slight non-significant increase of neuron numbers in the presence of IGF-1 could be observed. Although IGF-1 was able to increase the survival of mDA neurons in the presence of MPP^+^ (56.24% ± 10.39), the protective effect did not reach significance. Together, these results indicate that IL4 treatment of primary microglia results in the release of protective factors including IGF-1, which might at least partially mediate the neuroprotective properties of microglia-conditioned medium.

### Normal Morphology and Cellular Composition of the Nigrostriatal System in IL4-Deficient Mice

**FIGURE 4:**
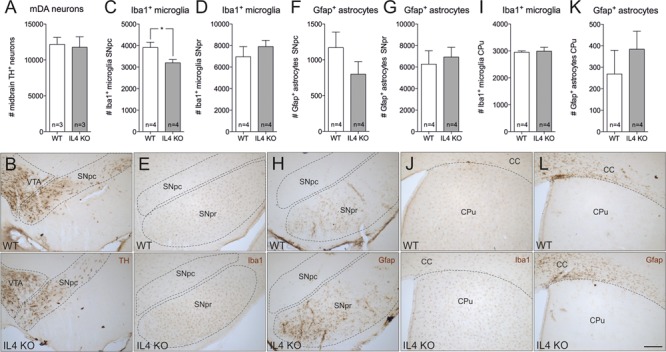
**Knockout of IL4 has no effect on the cellular composition of the nigrostriatal system.** Changes in numbers of TH^+^ neurons **(A,B)**, Iba1^+^ microglia **(C,D,E,I,J)** and Gfap^+^ astrocytes **(F,G,H,K,L)** in the nigrostriatal system were analyzed in 4 months old male WT and IL4 KO mice using unbiased stereology. A significant change in cell numbers was only detected for Iba1^+^ microglia in the SNpc, which were reduced in IL4 KO mice. Scale bar indicates 200 μm. Data are given as mean ± SEM from at least three animals per genotype. *P*-values derived from student’s *t*-test are ^∗^*p* < 0.05. VTA, ventral tegmental area; SNpc, substantia nigra, pars compacta; SNpr, substantia nigra, pars reticularis; CC, corpus callosum; CPu, caudate putamen.

In order to address the role of endogenous IL4 during MPTP-induced degeneration of mDA neurons *in vivo*, IL4-deficient mice were used. IL4 KO mice are viable and fertile, develop normally and show no phenotypical abnormalities. However, these mice show impaired secretion of IL5 and IL10 and reduced IL4-dependent expression of IgE and IgG1 (Metwali et al., 1996). As a prerequisite for the use of IL4 KO mice in the MPTP model, we first analyzed the cellular composition of the nigrostriatal system in adult mice. Therefore, 4 months old mice were perfused and brains were cut into 50 μm coronal sections, which were subsequently used for TH-, Iba1- and Gfap-immunohistochemistry. Cell numbers were determined using unbiased stereology. As shown in **Figures 4A,B**, the numbers of TH^+^ mDA neurons in the substantia nigra pars compacta (SNpc) were not altered in IL4-deficient mice. Quantification of Iba1^+^ microglia in SNpc and SNpr revealed a slight and significant decrease in microglia numbers in SNpc (**Figures 4C,E**) but no impairment of Iba1^+^ microglia numbers in SNpr (**Figures 4D,E**). Analysis of Gfap^+^ astrocytes showed that the numbers of reactive astrocytes are neither changed in the SNpc, nor in the SNpr (**Figures 4F–H**). Similar results were obtained to the basal ganglia (CPu). The numbers of Iba1^+^ microglia in the CPu of IL4 KO mice were comparable to WT mice (**Figures 4I,J**). Numbers of Gfap+ astrocytes in the CPu were increased in IL4 KO mice without reaching statistical significance (**Figures 4K,L**). These data indicate that loss of IL4 does not impair development and maintenance of the nigrostriatal system *in vivo*.

### Loss of IL4 Does Not Alter the Susceptibility toward MPTP-Induced Neurodegeneration

**FIGURE 5:**
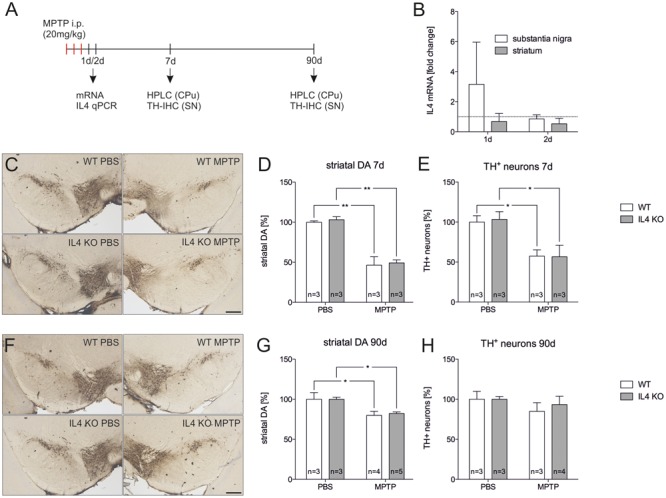
**Lack of IL4 has no impact on the susceptibility toward 1-methyl-4-phenyl-1,2,3,6-tetrahydropyridine (MPTP)-induced neurodegeneration. (A)** Scheme for MPTP injections and time points used for qPCR, HPLC and immunohistochemistry. **(B)** Expression of IL4 in total tissue samples from substantia nigra (SN) and caudate putamen (CPu) of wild type (WT) mice 1 and 2 days after MPTP injections. qPCR results were normalized to Gapdh and are given as fold changes (*n* = 3 PBS, *n* = 3 MPTP). **(C)** Immunohistochemical detection of TH^+^ neurons in the SN 7 days after injections with PBS and MPTP. Scale bar indicates 300 μm. **(D)** Striatal dopamine levels in PBS- and MPTP-injected mice after 7 days. Equal reductions in dopamine levels were observed in both genotypes. **(E)** Quantification of TH^+^ neuron numbers in the SN of PBS- and MPTP-injected mice. No significant changes in neurodegeneration were detected between WT and mutant (IL4 KO) mice. **(F)** Immunohistochemical detection of TH^+^ neurons in the SN 90 days after injections with PBS and MPTP. **(G)** Striatal dopamine levels in PBS- and MPTP-injected mice after 90 days. Similar recoveries of dopamine levels were observed in WT and IL4 KO mice. **(H)** TH^+^ neuron numbers in the SN of PBS- and MPTP-injected mice after 90 days were not significantly different between WT and IL4 KO mice, indicating normal regeneration of mDA neurons in IL4-deficient mice. Scale bars indicate 300 μm. Data are given as mean ± SEM from at least three mice per genotype and time point. *P*-values derived from one-way ANOVA followed by Bonferroni’s multiple comparison post-test are ^∗^*p* < 0.05 and ^∗∗^*p* < 0.01.

Since endogenous IL4 was sufficient to protect mDA neurons from MPP^+^-induced neurodegeneration *in vitro*, we used the MPTP mouse model to address the role of IL4 in this model for PD *in vivo*. **Figure 5A** displays the injection scheme and the time points used for RNA isolation, immunohistochemistry, and analysis of DA levels using HPLC. We first determined the expression of IL4 in the MPTP model. As shown in **Figure 5B**, a slight but not significant increase in IL4 expression was observed in the SN 1 day after MPTP injection. All other time points analyzed revealed no increases in IL4 expression in SN or CPu after intoxication with MPTP. Noteworthy, the relatively high CT values (between 32–36) indicate that in contrast to E14 mixed neuron-glia cultures, IL4 is hardly expressed in the nigrostriatal system *in vivo*. Two different time points for analysis of TH^+^ neuron numbers and striatal DA levels were chosen according to the acute neurodegeneration phase (7 days) and the regeneration phase (90 days) of this model (Machado et al., 2016c). **Figures 5C,E** demonstrate that numbers of TH^+^ neurons were significantly reduced in both WT (57.4% ± 7.895) and IL4 KO mice (56.67% ± 14.39) without showing significant differences between both genotypes. Similar results were obtained after analysis of striatal DA levels. IL4 KO mice displayed a significant reduction of DA levels (46.4% ± 10.51) comparable to WT mice (49.21% ± 3.72, **Figure 5D**). Analysis of neuron numbers and DA levels 90 days after MPTP administration revealed that WT and IL4 KO mice showed a similar restoration of the MPTP-intoxicated nigrostriatal system. As shown in **Figures 5F,H**, numbers of mDA neurons in the SNpc recovered similarly in WT (84.82% ± 10.86) as well as in IL4 KO mice (93.29% ± 10.4). Moreover, striatal DA levels increased in both genotypes (WT: 79.87% ± 4.956, IL4 KO: 82.14% ± 1.976) compared to 7 days after intoxication, but were still significantly reduced at 90 days compared to PBS-injected control mice (**Figure 5G**). Taken together, these data demonstrate that loss of endogenous IL4 does not increase the susceptibility toward MPTP-induced degeneration of mDA neurons *in vivo*. Moreover, the regeneration of the nigrostriatal system after MPTP administration is not impaired in IL4 KO mice, indicating that endogenous IL4 is dispensable in the MPTP mouse model for PD.

## Discussion

In the present study, we have demonstrated that exogenous IL4 protects mDA neurons from MPP^+^-induced degeneration in the presence of glia cells *in vitro*. Moreover, we provide evidence that endogenous microglia-derived IL4 in mixed neuron-glia cultures is important to reduce mDA neurodegeneration after MPP^+^ application and might facilitate neuroprotection by regulating microglia-mediated secretion of neuroprotective factors such as IGF-1. *In vivo*, the expression of IL4 was hardly detectable under basal conditions or after MPTP administration, and the loss of IL4 did not alter the susceptibility of mDA neurons toward MPTP-induced degeneration. Moreover, the regeneration of the nigrostriatal system after MPTP intoxication, as evident by restoration of TH^+^ neuron numbers as well as striatal DA levels, was not impaired in IL4-deficient mice.

Interleukin 4 has been described to promote a microglia activation state, which is characterized by functional features such as tissue repair and cellular protection (Colton and Wilcock, 2010). Here, we have demonstrated that microglial expression and secretion of cytokines/chemokines is not dramatically altered after IL4 treatment and thus, the neuroprotective effects for TH^+^ neurons seem to be mediated by other microglia-derived factors. However, IL4 has been reported to induce IL6-producing macrophages that inhibit neuroinflammation *in vitro* and *in vivo* (Casella et al., 2016). Interestingly, IL6 is able to protect mDA neurons from MPP^+^-driven degeneration (Spittau et al., 2012) and reduced expression of IL6 has been linked to increased degeneration of mDA neurons after treatment of GDF15-deficient mice with 6-OHDA (Machado et al., 2016b). Nevertheless, IL6 was not regulated after treatment of microglia with IL4 in the cell culture model used in this study. Another molecular mechanism to explain IL4-mediated neuroprotection in mixed neuron-glia cultures is the regulation of microglia function via the CD200/CD200R ligand/receptor pair. Whereas neurons express CD200, expression of CD200R is restricted to myeloid cells including microglia and binding of CD200 to its cognate receptor reduces microglia activation (Barclay et al., 2002). IL4 has been shown to modulate these interactions by increasing the surface expression of CD200R in microglia (Lyons et al., 2007). Moreover, lack of CD200 results in increased microglia activation after facial nerve transection (Hoek et al., 2000) indicating the importance of this neuron-microglia communication. However, in the present study we observed that microglial conditioned medium after IL4 stimulation is neuroprotective without the physical presence of microglia. This observation suggests that microglia secrete neurotrophic factors after IL4 treatment, which is at least partially responsible for the protection of mDA neurons. We identified IGF-1 as one of these neurotrophic factors and Wynes et al. (2004) have described IL4-mediated upregulation of IGF-1 in macrophages. Interestingly, IGF-1 has been reported to protect mDA neurons in PD models *in vivo*. IGF-1 increased survival of TH^+^ neurons and reduced neuroinflammation after intoxication with MPTP (Nadjar et al., 2009) as well as after 6-OHDA injections (Guan et al., 2000). We further tried to rescue mDA neurons from MPP^+^-induced degeneration, by adding recombinant IGF-1 to neuron-enriched cultures. Although the number of surviving neurons increased, the protection was not as effective as IL4 treatment of mixed neuron-glia cultures. It is high likely that microglia-derived neurotrophic factors and neuron–microglia interactions contribute to the neuroprotective effects observed after IL4 application in neuron-glia cultures. The fact that IL4 secretion from microglia is not a direct effect of MPP^+^ suggests that challenged neurons in mixed neuron-glia cultures trigger IL4 release from microglia. Damaged neurons have been shown not be passive targets of microglia-mediated neurotoxicity but are capable of regulating microglia functions. Release of CX3CL1 or IL34 from neurons and the subsequent binding to microglia-specific receptors CX3CR1 and CSF1R are well described mechanisms to shape microglia functions toward a neuroprotective phenotype (Suzumura, 2013). Such neuron–microglia interactions might also be responsible for the IL4 release observed in mixed-neuron glia cultures in the present study and should be further analyzed to understand how MPP^+^-challenged neurons regulate microglia functions.

*In vitro*, neutralization of endogenous IL4, expressed by microglia, worsens MPP^+^-induced degeneration of mDA neurons. This result suggests that IL4 acts in an autocrine manner on microglia in order to silence their activation. *In vivo*, loss of IL4 did not change the extent of neurodegeneration and neuroregeneration, indicating that IL4 is dispensable in the MPTP mouse model for PD. One of the reasons for these observations, might be the fact that IL4 expression in the nigrostriatal system is hardly detectable under basal conditions as well as after MPTP intoxication. The main cell type expressing IL4 are Th2 T-cells (Bao and Reinhardt, 2015). However, myeloperoxidase-positive neutrophils after spinal cord injury (Lee et al., 2010) and also damaged neurons (Zhao et al., 2015) are able to express IL4 under neuropathological conditions. The MPTP mouse model for PD is characterized by a distinct leakage of the blood-brain-barrier (BBB) and a modest infiltration of CD3^+^ T-cells (Chung et al., 2013) but the neuroinflammatory response is predominantly performed by brain-resident microglia (Depboylu et al., 2012). This is in contrast to the BBB impairment observed in the 6-OHDA mouse model for PD, where a pronounced invasion of T-cells and B-cells have been described which makes this model more suitable to study the contribution of adaptive immune responses during mDA degeneration (Theodore and Maragos, 2015). Lack of appropriate numbers of IL4-expressing T-cells might explain why the extent of mDA neurodegeneration induced by MPTP was not altered in IL4-deficient mice used in the present study. The potential of CD4^+^ T-cells to protect mDA neurons from MPTP-induced neurodegeneration has been described after adoptive transfer of CD4^+^/CD25^+^ regulatory T-cells (Reynolds et al., 2007) and highlight the therapeutic options of these anti-inflammatory cells. It would be of major interest to address the role of endogenous IL4 in the 6-OHDA mouse model for PD, where the involvement of adaptive immune responses has been clearly demonstrated. Moreover, it has to be taken into account that alternative microglia activation in PD models as evidenced by expression of M2-like markers such as Ym1 and Arg1 (Haas et al., 2016) might be regulated by IL4-independent mechanisms. Contact to apoptotic cells and subsequent release of microglial TGFβ1 and TGFβ2 resulting in increased expression of the M2-like marker Arg1 has been described (Spittau et al., 2015). Microglial TGFβ-signaling seems to be essential for promoting alternative microglia activation (Zhou et al., 2012) and could be one mechanism to trigger IL4-independent M2-like microglia activation.

The therapeutic potential of exogenous IL4 has been well documented in several CNS pathology models including models for spinal cord injury (Lee et al., 2010; Francos-Quijorna et al., 2016), APP/PS1 transgenic mice (Kiyota et al., 2010), cerebral ischemia (Zhao et al., 2015; Liu et al., 2016) as well as multiple sclerosis (Butti et al., 2008). In these studies, IL4 was either injected or overexpressed in CNS tissues and promoted strong neuroprotective and anti-inflammatory effects. It has been shown that a heterogeneous induction of the microglia M2a phenotype was observed after central administration of IL4 and contributes to neuroprotection (Pepe et al., 2014). However, further studies have to be performed in order to determine the potential of exogenous IL4 in animal models for PD including MPTP and 6-OHDA.

Taken together, the results presented in this study demonstrate that IL4 is able to shape microglia functions to promote survival of mDA neurons. We provided evidence that endogenous IL4 is dispensable for MPTP-induced neurodegeneration *in vivo* but the presence of IL4 *in vitro* is important to limit degeneration of TH^+^ neurons after MPP^+^ intoxication. Our results strengthen the hypothesis that IL4-treated microglia are capable of facilitating protection of mDA neurons and underline the therapeutic potential of IL4 administration in models of PD.

## Author Contributions

BS conceived the project. LH, JR, XZ, VM, and BS performed experiments and analyzed the data. RG performed HPLC analyses. BS wrote the manuscript. All authors have read and approved the final manuscript and further agreed to be accountable for the content of the work.

## Conflict of Interest Statement

The authors declare that the research was conducted in the absence of any commercial or financial relationships that could be construed as a potential conflict of interest.
